# A Fatal Case of Multidrug Resistant *Acinetobacter* Necrotizing Fasciitis: The Changing Scary Face of Nosocomial Infection

**DOI:** 10.1155/2014/705279

**Published:** 2014-10-02

**Authors:** Nupur Sinha, Masooma Niazi, Dmitry Lvovsky

**Affiliations:** ^1^Division of Pulmonary and Critical Care Medicine, Bronx Lebanon Hospital Center, Albert Einstein College of Medicine, Bronx, NY 10457, USA; ^2^Albert Einstein College of Medicine, Department of Pathology, Bronx Lebanon Hospital Center, Bronx, NY 10457, USA

## Abstract

Necrotizing fasciitis is an uncommon soft-tissue infection, associated with high morbidity and mortality. Early recognition and treatment are crucial for survival. *Acinetobacter baumannii* is rarely associated with necrotizing fasciitis. Wound infections due to *A. baumannii* have been described in association with severe trauma in soldiers. There are only sporadic reports of monomicrobial *A. baumannii* necrotizing fasciitis. We report a unique case of monomicrobial necrotizing fasciitis caused by multidrug resistant (MDR) *A. baumannii*, in absence of any preceding trauma, surgery, or any obvious breech in the continuity of skin or mucosa. A 48-year-old woman with history of HIV, asthma, hypertension, and tobacco and excocaine use presented with acute respiratory failure requiring mechanical ventilation. She was treated for pneumonia for 7 days and was successfully extubated. All septic work-up was negative. Two days later, she developed rapidly spreading nonblanching edema with bleb formation at the lateral aspect of right thigh. Emergent extensive debridement and fasciotomy were performed. Operative findings and histopathology were consistent with necrotizing fasciitis. Despite extensive debridement, she succumbed to septic shock in the next few hours. Blood, wound, and tissue cultures grew *A. baumannii*, sensitive only to amikacin and polymyxin. Histopathology was consistent with necrotizing fasciitis.

## 1. Introduction

Necrotizing fasciitis is an uncommon soft-tissue infection and is associated with high morbidity and mortality. Timely clinical diagnosis is challenging and early recognition and treatment are the most important factors affecting survival. First described by a Confederate Army Surgeon, Joseph Jones, during the US Civil War [1] and subsequently by Fournier in the scrotum and penis [2, 3] and by Meleney in a series of patients with streptococcal disease [4], it is a polymicrobial infection in approximately 70% of cases [5, 6]. The most common pathogens associated with necrotizing fasciitis include group A, beta-hemolytic* Streptococcus*, group B,* Streptococcus*,* Enterococci*,* Coagulase-negative Staphylococci, Staphylococcus aureus, Escherichia coli, Pseudomonas aeruginosa, Bacteroides, and Clostridium *[7].* Acinetobacter baumannii *is rarely associated with necrotizing fasciitis, especially in immunocompetent hosts [6, 8]. We report an unusual case of a female with rapidly developing fatal* A. baumannii *associated necrotizing fasciitis.

## 2. Case Report

**Figure 1:**
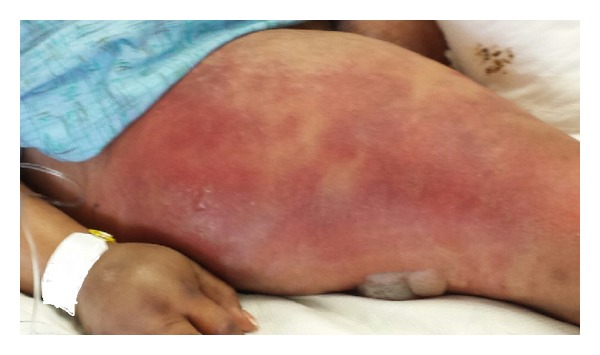
Erythematous rash with nonblanching edema, bleb formation, skin peeling, and areas of bogginess at the lateral aspect of right thigh.

**Figure 2:**
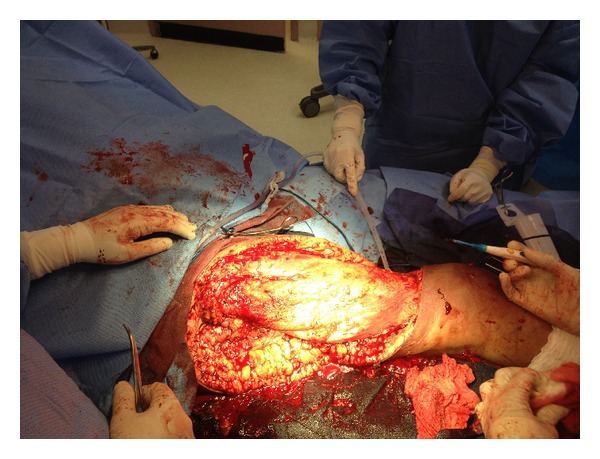
Surgical debridement revealing extensive area of necrotizing fasciitis on anterior, medial, and lateral right thigh involving epidermis, dermis, subcutaneous tissue, and fascia.

**Figure 3:**
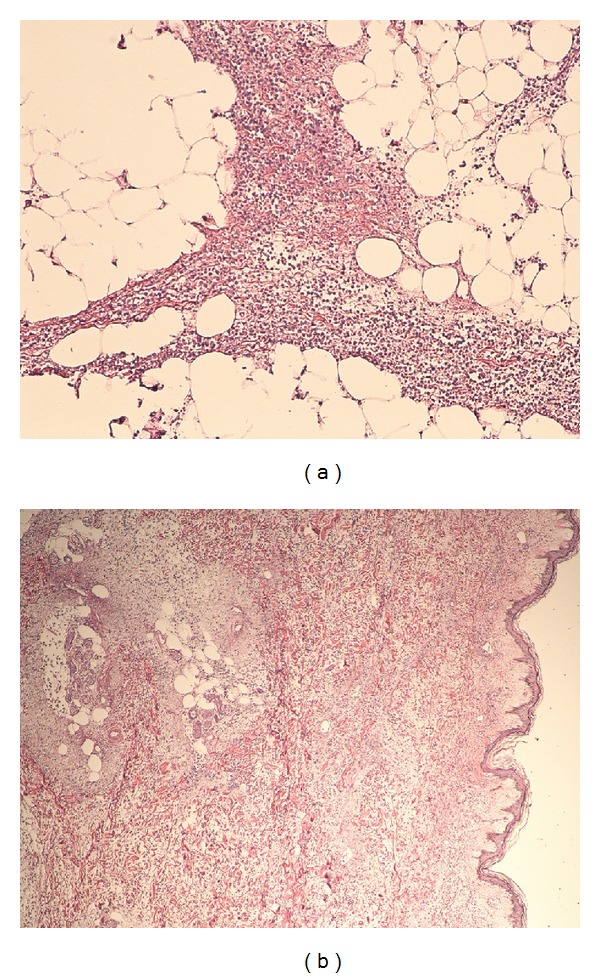
Histopathology: (a) subcutaneous fat with marked acute inflammatory cell infiltrate, comprised of polymorphs, macrophages, and cellular debris; (b) acute inflammation with scattered necrosis and vascular microthrombosis involving dermis, subcutaneous fat, and fascia.

We report a 48-year-old woman admitted with altered mental state with preceding history of productive cough and shortness of breath for 3 days. There was no history of fever, chills, sick contact, hemoptysis, recent travel, trauma, or weight loss. Her medical history included HIV (CD4–47), intermittent bronchial asthma, hypertension, mood disorder, and HIV associated neuropathy. She was an active tobacco smoker with approximately 20-pack-year history and excocaine user. She had no significant family history. She was allergic to penicillin. Physical examination revealed fever with temperature 102.3 F, tachycardia with heart rate 135 bpm, hypoxia with oxygen saturation at 84%, and hypotension with systolic blood pressure of 88 mm Hg. Laboratory parameters indicated respiratory and metabolic acidosis (pH 7.18, pCO_2_ 66, bicarbonate 17), chronic unchanged anemia (hemoglobin 8.7 gm/dL) and mild transaminitis (SGOT 47 U/L), baseline creatinine (1.5 mg/dL), and absence of any leukocytosis or leucopenia. Urinalysis was normal. Urine and blood cultures were sent. CXR (chest X-ray) was suggestive of bibasilar opacities. Initial hypotension resolved on volume resuscitation via peripheral lines placed in emergency room. She was soon intubated for hypoxic-hypercapnic respiratory failure. Intubation was uneventful and bronchoscopy revealed normal mucosa and anatomy with thick mucoid secretions. She was started on vancomycin, aztreonam, and amikacin to treat presumed healthcare associated pneumonia while awaiting BAL (bronchoalveolar lavage) results and other septic workup. Amikacin was discontinued by day 3 as the cultures remained sterile. Her blood and urine cultures from the day of admission did not show any microbial growth. She was continued on vancomycin and aztreonam for presumptive pneumonia. On the 7th day, vancomycin and aztreonam were discontinued after BAL cultures reported growth of only parainfluenza 3. She was liberated from mechanical ventilation and successfully extubated on the 10th day. She remained afebrile, although, noted to be in delirium. On the 12th day she had a temperature spike of 102 F and was restarted on vancomycin and aztreonam. In next 4 hrs she was noted to have an erythematous (approximately 3 cm in diameter) rash on lateral aspect of her right upper thigh. In next 2-3 hrs she developed rapidly spreading erythema and nonblanching edema, bleb formation, skin peeling, and areas of bogginess at the lateral aspect of thigh (Figure 1). Distal pulses were intact. Patient had no peripheral or central line placement in the groin or any blood draws from the lower extremities during the hospital course, and all her medications were being administered via a peripherally placed central catheter in right upper extremity. Presumptive diagnosis of cellulitis versus possible necrotizing fasciitis was made. She was reintubated, intravenous clindamycin was added to her existing regime, and she was emergently taken to operating room for extensive debridement and possible fasciotomy. On exploration she was noted to have extensive area of necrotizing fasciitis on anterior, medial, and lateral right thigh totaling 35 × 25 cm, involving epidermis, dermis subcutaneous tissue, and portion of fascia. There was no evident muscle involvement. A wide debridement of the dead tissue (Figure 2) was performed with an estimated blood loss of 1000 mL intraoperatively. She received blood, platelet, and FFP transfusions during the procedure in an effort to achieve maximal debridement. She had a rapidly fatal course over next few hours requiring vasopressor support with extension of cellulitis distally to right foot and proximally to lower abdominal wall with crepitus and blisters. Despite aggressive resuscitative measures, she succumbed to septic shock in less than 20 hrs from the time of the clinical diagnosis. Blood cultures drawn at this time were later reported to grow* A. baumannii,* sensitive only to amikacin and polymyxin. All prior blood cultures had been negative. Wound cultures reported heavy growth of* A. baumannii,* sensitive only to amikacin and polymyxin. Tissue cultures from debrided right thigh also showed heavy growth of* A. baumannii, *sensitive to amikacin, ampicillin/sulbactam, polymyxin, and tobramycin. Histopathology revealed severe acute inflammation and focal tissue necrosis involving epidermis, dermis and subcutaneous tissue, and portion of fascia, consistent with necrotizing fasciitis (Figure 3).

## 3. Discussion

Necrotizing fasciitis is a rare soft tissue infection, with annual age-adjusted incidence of approximately 4.3 infections per 100,000 of the US population [9]. It is characterized by rapidly progressive necrosis of fascia and subcutaneous tissue with relative sparing of underlying muscle [6, 9–11]. The fulminant tissue necrosis may rapidly evolve into septic shock and multiorgan failure. Early recognition is usually difficult and has rapidly fatal course in absence of prompt aggressive management [11]. The initial event in the etiopathogenesis is the entry of bacteria into the fascia, spontaneously or secondary to either trauma or surgery [9, 12]. Rapid bacterial proliferation in the fascia is followed by leukocytic infiltration and liquefactive necrosis. Subsequent progressive thrombosis of the blood vessels in the fascia leads to occlusion of the perforating skin vessels and secondary cutaneous ischemia and gangrene [9]. Known to be associated with mortality as high as 75% [9, 11, 13], necrotizing fasciitis is a surgical emergency and prompt diagnosis is crucial. Patients usually present with the triad of excruciating pain out of proportion to physical findings, fever, and area of erythema and warmth [11]. Presence of bullae filled with serous fluid is an important diagnostic clue and should raise the index of suspicion [11, 13]. Prompt aggressive surgical debridement of all necrotic tissue is of utmost importance due to ineffective antibiotic delivery to the involved area in presence of thrombosis of supplying blood vessels [11] and is known to improve the survival rate [11, 14]. It is classically described in 2 subtypes. Type-I infections are polymicrobial and account for the most common 55% to 75% of cases. Type-II infections result from group A* Streptococcus* either alone or in association with* Staphylococcus aureus *[6]. The most commonly identified microorganisms are group A and B streptococci, the staphylococci, members the family* Enterobacteriaceae*, and* Pseudomonas aeruginosa* [6, 12, 15]. Some reports also describe type-III infections, those caused by* Vibrio vulnificus *[6, 16].

*A. baumannii, *an aerobic, gram-negative bacillus, ubiquitously isolated from soil, water, sewage, and healthcare settings, is being recognized as an increasingly prevalent and significant pathogen [5, 12, 17]. Often associated with immunocompromised and hospitalized patients, it is well described in cases of sepsis, wound infections, and pneumonia in these populations [5]. Rarely,* A. baumannii* has been associated with community-acquired bacteremic cellulitis and community-acquired pneumonia in previously healthy people [8, 18]. Wound infections due to* A. baumannii *have been classically described in association with severe trauma, as in American soldiers wounded in Iraq and Afghanistan [5, 19]. The increasing incidence of antibiotic resistance has highlighted the importance of* A. baumannii* as nosocomial pathogen [12, 20]. Despite its increasing prevalence and capacity to express resistance to multiple classes of antibiotics, surveillance efforts indicate only rare involvement of* A. baumannii *in skin and soft tissue infections [21, 22]. Although* A. baumannii *has been reported as a copathogen in polymicrobial necrotizing fasciitis in severely ill and immunocompromised patients [5, 6, 19, 21, 23], there are only sporadic case reports of monomicrobial association (Table 1). An earlier review of 87 patients had reported* A. baumannii *as a monomicrobial cause of necrotizing fasciitis in 2 patients [23], but the characteristics of these patients are not clear and authors acknowledged “relatively unsophisticated techniques for collection, transfer, and culture of anaerobic specimens” to be responsible for reported overall high rate of monomicrobial infection [5].

Extensive review of literature reveals only five cases implicating* A. baumannii *as the monomicrobial cause of necrotizing fasciitis where specimens were collected and incubated reliably (Table 1). All cases were reported in the setting of trauma or instrumentation. The cellulitis and skin breakdown of left upper extremity on recent hospitalization provided the likely entry site in patient 1. Patients 2 and 3 were reported in the end-stage renal disease patients with hemodialysis access and developed necrotizing fasciitis in the immediate postlaparotomy period for underlying gastrointestinal and genitourinary tract infections. While blood and tissue cultures grew only* A. baumannii*, their earlier hospital course was complicated by polymicrobial infection of other organs and had received multiple antibiotics, thus contributing to the resistant strain of* A. baumannii *isolate. The association with war trauma as in patients 4 and 5 is well described, mostly polymicrobial, and is associated with favorable prognosis [17, 19].

Our patient is a unique case of monomicrobial necrotizing fasciitis caused by multidrug resistant (MDR)* A. baumannii*, in absence of any preceding trauma, surgery, or any obvious breech in the continuity of skin or mucosa. Preceding multiple cultures had been negative and she had received only 7 days of antibiotics. The risk factor in our case was immune-compromised status secondary to HIV, thus making her susceptible to any healthcare associated infection.* Acinetobacter *spp. are reportedly the most common gram-negative bacteria to colonize the skin of hospital personnel, but even among immune-compromised patients, the pathogen does not usually cause necrotizing fasciitis [5, 24]. It is commonly associated with bloodstream, lung, or milder skin and soft tissue infections. The most unusual thing about our case was absence of any inciting trauma or surgery and MDR* A. baumannii *being the sole isolate from multiple tissue cultures, in absence of any copathogen or prolonged hospital course.

MDR* A. baumannii *strains have been defined as* A. baumannii *strains resistant to three or more representatives of the following classes of antibiotics: fluoroquinolones (ciprofloxacin), extended-spectrum cephalosporins (ceftazidime and cefepime), lactam-lactamase inhibitor combinations (ampicillin-sulbactam), aminoglycosides (amikacin and tobramycin), and carbapenems (imipenem and meropenem) [5].

Despite ever-increasing population of immune-compromised and hospitalized patients with multiple comorbidities, the entity described here has not been reported earlier.

## 4. Conclusion

Our case brings forth the issue of increasing prevalence of MDR* A. baumannii *strains and its varied and scary presentations. The* Acinetobacter* association shown earlier among soldiers has not been associated with significant mortality. In our case, despite fairly timely recognition and aggressive debridement, the rapidly fatal course could not be altered. Absence of any inciting trauma, surgery, or preceding prolonged course in our case reveals the changing scary face of nosocomial infection and warrants low threshold for recognition of this entity and timely intervention. As illustrated in our case infection with MDR* A. baumannii* can be rapidly fatal, thus stressing the importance of reporting* A. baumannii *isolates in cases of necrotizing fasciitis. Physicians should be aware of the remarkable virulence and drug resistance that these ubiquitous colonizers can acquire in unsuspecting soft tissue infection. Historically, even expert clinicians would not expect* A. baumannii *to be the primary etiological agent causing fatal necrotizing fasciitis. The reporting of confirmed case of MDR* A. baumannii* associated necrotizing fasciitis warrants consideration for expanding antibiotic coverage to include this pathogen.

## Figures and Tables

**Table 1:** Patient characteristics of reported monomicrobial *Acinetobacter baumannii* associated necrotizing fasciitis.

Patient [reference]	Age	Sex	Comorbidities	Devices present	Location	Sepsis	*Acinetobacter* culture site	Surgery/trauma	Prior cultures	Prior use of antibiotics	Outcome
1 [12]	83	M	CAD, CHF, cirrhosis, left upper arm cellulitis (4 weeks ago)	No	Left upper arm	Yes	Blood	Unknown	Unknown	Yes	Died

2 [5]	21	M	SLE, ESRD, TTP, mesenteric vasculitis	Yes	Left flank and thigh	Yes	Blood & muscle tissue	Yes	*E. faecium*, *Candida albicans*, *Clostridium difficile*, *K. pneumoniae*, *Citrobacter freundii *	Multiple	Died within 36 h of initial symptom

3 [5]	47	F	HIV, ESRD	Yes	Right thigh	Yes	Blood	Yes	Methicillin resistant coag. neg. staph, *P. aeruginosa *	Multiple	Died in 18 days

4 [19]	55	M	Gunshot wound on right buttock, femur fracture, sciatic nerve injury	Yes	Hip, abdomen	Yes	Blood, OR cultures	Yes	None	Multiple	Survived

5 [19]	22	M	Gunshot wound on abdomen	Yes	Abdomen, flank	Yes	Blood, bullae	Yes	None	Multiple	Survived

Index patient	48	F	HIV, asthma, HTN, mood disorder	No	Right thigh and flank	Yes	Blood, debrided tissue	No	None	Yes	Died within 20 h of initial symptom

CAD: coronary artery disease; CHF: congestive heart failure; ESRD: end-stage renal disease; HIV: human immunodeficiency virus; SLE: systemic lupus erythematosus; TTP: thrombotic thrombocytopenic purpura.

## Abbreviations

BAL: Bronchoalveolar lavage
CXR: Chest X-ray
HIV: Human immunodeficiency virus
MDR: Multidrug resistant.

## References

[1] Quirk WF, Sternbach G (1996). Joseph Jones: infection with flesh eating bacteria. *The Journal of Emergency Medicine*.

[2] Smith GL, Bunker CB, Dinneen MD (1998). Fournier's gangrene. *British Journal of Urology*.

[3] Fournier A (1883). Gangrène foudroyante de la verge. *La Semaine Médicale*.

[4] Meleney FL (1924). Hemolytic streptococcus gangrene. *Archives of Surgery*.

[5] Charnot-Katsikas A, Dorafshar AH, Aycock JK, David MZ, Weber SG, Frank KM (2009). Two cases of necrotizing fasciitis due to *Acinetobacter baumannii*. *Journal of Clinical Microbiology*.

[6] Corradino B, Toia F, di Lorenzo S, Cordova A, Moschella F (2010). A difficult case of necrotizing fasciitis caused by *Acinetobacter baumannii*. *International Journal of Lower Extremity Wounds*.

[7] Elliott D, Kufera JA, Myers RAM (2000). The microbiology of necrotizing soft tissue infections. *The American Journal of Surgery*.

[8] Chiang W-C, Su C-P, Hsu C-Y (2003). Community-acquired bacteremic cellulitis caused by *Acinetobacter baumannii*. *Journal of the Formosan Medical Association*.

[9] Castleberg E, Jenson N, Dinh VA (2014). Diagnosis of necrotizing faciitis with bedside ultrasound: the staff exam. *Western Journal of Emergency Medicine*.

[10] Patino JF, Castro D (1991). Necrotizing lesions of soft tissues: a review. *World Journal of Surgery*.

[11] Wong C-H, Chang H-C, Pasupathy S, Khin L-W, Tan J-L, Low C-O (2003). Necrotizing fasciitis: clinical presentation, microbiology, and determinants of mortality. *Journal of Bone and Joint Surgery Series A*.

[12] Sullivan DR, Shields J, Netzer G (2010). Fatal case of multi-drug resistant *Acinetobacter baumannii* necrotizing fasciitis. *The American Surgeon*.

[13] Weiss KA, Laverdière M (1997). Group A *Streptococcus* invasive infections: a review. *Canadian Journal of Surgery*.

[14] Voros D, Pissiotis C, Georgantas D, Katsaragakis S, Antoniou S, Papadimitriou J (1993). Role of early and extensive surgery in the treatment of severe necrotizing soft tissue infection. *British Journal of Surgery*.

[15] Swartz MN, Pasternack MS, Mandell GL, Bennett JE, Dolin R (2010). Cellulitis, necrotizing fasciitis, and subcutaneous tissue infections. *Principles and Practice of Infectious Diseases*.

[16] Sarani B, Strong M, Pascual J, Schwab CW (2009). Necrotizing fasciitis: current concepts and review of the literature. *Journal of the American College of Surgeons*.

[17] Dallo SF, Weitao T (2010). Insights into acinetobacter war-wound infections, biofilms, and control. *Advances in Skin & Wound Care*.

[18] Chen M-Z, Hsueh P-R, Lee L-N, Yu C-J, Yang P-C, Luh K-T (2001). Severe community-acquired pneumonia due to *Acinetobacter baumannii*. *Chest*.

[19] Sebeny PJ, Riddle MS, Petersen K (2008). Acinetobacter baumannii skin and soft-tissue infection associated with war trauma. *Clinical Infectious Diseases*.

[20] Zeana C, Larson E, Sahni J, Bayuga SJ, Wu F, Della-Latta P (2003). The epidemiology of multidrug-resistant *Acinetobacter baumannii*: does the community represent a reservoir?. *Infection Control and Hospital Epidemiology*.

[21] Guerrero DM, Perez F, Conger NG (2010). Acinetobacter baumannii-associated skin and soft tissue infections: recognizing a broadening spectrum of disease. *Surgical Infections*.

[22] Moet GJ, Jones RN, Biedenbach DJ, Stilwell MG, Fritsche TR (2007). Contemporary causes of skin and soft tissue infections in North America, Latin America, and Europe: Report from the SENTRY Antimicrobial Surveillance Program (1998–2004). *Diagnostic Microbiology and Infectious Disease*.

[23] Liu Y-M, Chi C-Y, Ho M-W (2005). Microbiology and factors affecting mortality in necrotizing fasciitis. *Journal of Microbiology, Immunology and Infection*.

[24] Larson EL (1981). Persistent carriage of gram-negative bacteria on hands. *American Journal of Infection Control*.

